# Quality indicators in intensive care medicine for Germany – third edition 2017

**DOI:** 10.3205/000251

**Published:** 2017-08-01

**Authors:** Oliver Kumpf, Jan-Peter Braun, Alexander Brinkmann, Hanswerner Bause, Martin Bellgardt, Frank Bloos, Rolf Dubb, Clemens Greim, Arnold Kaltwasser, Gernot Marx, Reimer Riessen, Claudia Spies, Jörg Weimann, Gabriele Wöbker, Elke Muhl, Christian Waydhas

**Affiliations:** 1 Department of Anesthesiology and Intensive Care Medicine, Charité – Universitätsmedizin Berlin, corporate member of Freie Universität Berlin, Humboldt-Universität zu Berlin, and Berlin Institute of Health, Berlin, Germany; 2 Department of Anesthesiology and Intensive Care Medicine, Martin-Luther Krankenhaus, Berlin, Germany; 3 Department of Anaesthesiology and Intensive Care Medicine, Klinikum Heidenheim, Germany; 4 Department of Anaesthesiology and Intensive Care Medicine, Asklepiosklinikum Altona, Hamburg, Germany; 5 Department of Anaesthesiology and Intensive Care Medicine, St. Josef-Hospital, Klinikum der Ruhr-Universität Bochum, Germany; 6 Department of Anaesthesiology and Intensive Care Medicine, Jena University Hospital, Jena, Germany; 7 Kreiskliniken Reutlingen, Deutsche Gesellschaft für Fachkrankenpflege und Funktionsdienste (DGF), Germany; 8 Department of Anaesthesiology and Intensive Care Medicine, Klinikum Fulda, Germany; 9 Department of Intensive Care Medicine, Universitätsklinikum RTWH Aachen, Germany; 10 Zentralbereich des Departments für Innere Medizin, Internistische Intensivmedizin, Universitätsklinikum Tübingen, Germany; 11 Department of Anesthesiology and Interdisciplinary Intensive Care Medicine, Sankt Gertrauden-Krankenhaus, Berlin, Germany; 12 Department of Intensive Care Medicine, Helios-Klinikum Wuppertal, Germany; 13 Department of Surgery, Medical University of Schleswig Holstein, Kiel, Germany; 14 Department of Surgery, Berufsgenossenschaftliches Universitätsklinikum Bergmannsheil, Bochum, Germany; 15 Medical Faculty of the University Duisburg-Essen, Germany

**Keywords:** quality management, intensive care medicine, quality indicators, peer review

## Abstract

Quality improvement in medicine is depending on measurement of relevant quality indicators. The quality indicators for intensive care medicine of the German Interdisciplinary Society of Intensive Care Medicine (DIVI) from the year 2013 underwent a scheduled evaluation after three years. There were major changes in several indicators but also some indicators were changed only minimally. The focus on treatment processes like *ward rounds*, *management of analgesia and sedation*, *mechanical ventilation* and *weaning*, as well as the number of 10 indicators were not changed. Most topics remained except for *early mobilization* which was introduced instead of *hypothermia following resuscitation*. *Infection prevention* was added as an outcome indicator. These quality indicators are used in the peer review in intensive care, a method endorsed by the DIVI. A validity period of three years is planned for the quality indicators.

## Zusammenfassung

Qualitätsverbesserung in der Medizin hängt von der Messung relevanter Qualitätsindikatoren ab. Die Qualitätsindikatoren für die Intensivmedizin der Deutschen Interdisziplinären Vereinigung für Intensivmedizin (DIVI) aus dem Jahre 2013 sind nun planmäßig nach drei Jahren überarbeitet und angepasst worden. Hierbei gab es deutliche Änderungen in einigen Indikatoren während einige nur minimal verändert wurden. Der Fokus auf intensivmedizinische Behandlungsprozesse wie *interprofessionelle Visiten*, *Management von Analgesie und Sedierung*, *Beatmung* und *Weaning* blieb ebenso bestehen wie die Gesamtzahl von 10 Indikatoren. Die Themenschwerpunkte blieben weitgehend bestehen außer der *Frühmobilisation*, welche anstatt der *Hypothermie nach Reanimation* eingeführt wurde. I*nfektionsprävention* wurde als Ergebnisindikator hinzugefügt. Diese Qualitätsindikatoren werden im Peer Review Prozess der DIVI eingesetzt. Eine Gültigkeitsdauer von drei Jahren ist für diese Qualitätsindikatoren geplant.

## Introduction

In 2010 the first quality indicators (QI) in intensive care medicine were published for Germany by the German Interdisciplinary Society for Intensive Care Medicine (DIVI) [1]. Following an updated second edition in 2013 [2], this third edition of the quality indicators was developed that included some major changes. 

**Figure 1:**
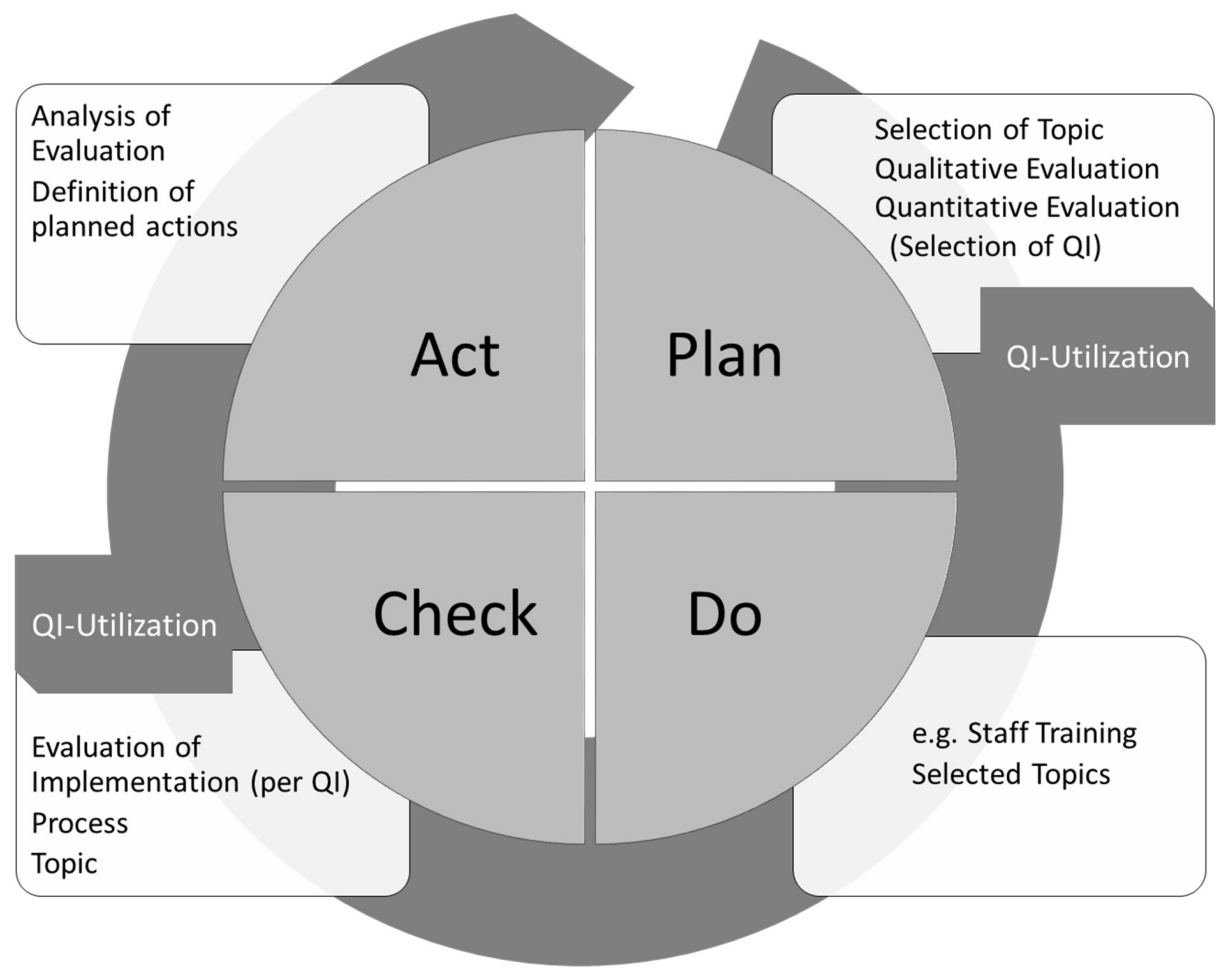
Introduction of quality indicators Use of quality indicators in intensive care medicine by employing the PDCA cycle. QI are useful for measurement of the actual situation to support further planning. Their main use is the evaluation of the effects of newly introduced actions. They provide the crucial link between “Check” and “Act”.

The quality indicators are an integral part of the peer review in intensive care medicine endorsed by the DIVI that has gained increasing acceptance and is now an established method of quality management [3], [4]. Analyzes from these peer reviews show that the implementation of QI is an important part of quality efforts in many intensive care units (ICUs). Even positive economic effects have been reported [5]. However, compared to other quality relevant topics, such as staffing and organization, the QI are still considered less relevant, are insufficiently distributed and not adequately reported [3]. The QI also receive attention at the political level, and some of the indicators are increasingly viewed as criteria for external quality assurance. This is an overall positive development. However, central aspects of the indicators should not be instrumentalized for economic purposes, i.e. reimbursement. They are primarily developed to describe topics critical for quality in intensive care medicine and then be further evaluated in peer reviews or on a local level. The aim is to enter a cycle of continuous improvement as shown in Figure 1 (Fig. 1). The QI are developed interdisciplinary and interprofessionally with a main focus of quality evaluation. The QI in principle do not represent minimal requirements for ICUs but may be interpreted as that. From our point of view further dissemination and especially implementation of the indicators is the priority for the coming years. The increased use of the QI shows that the requirements of the RUMBA rule are met:

- **R**elevant to the problem
- **U**nderstandable
- **M**easurable, with high reliability and validity
- **B**ehaviorable (changeable by behavior)
- **A**chievable (reachable and feasible).

Another important factor is the manageable number of ten indicators. The most relevant core processes of intensive care such as mechanical ventilation, analgesia, sedation and delirium treatment, anti-infective therapy, nutrition, hygiene, and communication with patients and relatives are represented. In addition, there is the structure criterion of staff qualification of the intensive care unit. Without these and other measures, quality and its improvement cannot be described adequately. Promoting quality in every day care is the goal of these quality indicators that are implemented in the peer review process.

## Development of the third edition of the quality indicators in intensive care medicine

As scientific evidence changes over time, it is necessary to review process based quality indicators regularly and adapt them if necessary [6]. The national steering group for the peer review in intensive care medicine has been assigned by the DIVI to revise the quality indicators on a regular basis with regard to underlying guidelines and new evidence in the literature. This latest revision process was started in September 2015. Initially, the medical societies organized in the DIVI were asked to submit proposals for new or revised quality indicators. In April 2016 these proposals from the societies were brought together. The list of new proposals was edited by the national steering committee and then discussed in a Delphi process. After consultation within the DIVI-affiliated societies, the quality indicators were confirmed in February 2017 by the council of the DIVI and approved for publication in June 2017.

## The quality indicators in intensive care medicine – comparison to other quality measures

A task force of the European Society of Intensive Care Medicine (ESICM) has published a list of quality indicators in 2012 [7]. These included structure indicators such as compliance with national standards and an “adverse event” reporting system. As process indicators, routine multidisciplinary visits in the intensive care unit and a standardized transfer protocol were consented. Outcome indicators included the standardized mortality rate (SMR) and the 48-hour re-admission rate, the rate of catheter-associated blood stream infections and the rate of unplanned extubations. In contrast the DIVI quality indicators comprise mainly process indicators which are used in the peer review process [4], [8]. Compared to the DIVI-QIs, outcome indicators are mainly used in seven other countries and by the ESICM [9]. Outcome indicators are a part of the German intensive medical core data set DIVI-Reversi (SMR and 48h resuscitation rates). The “adverse event” indicator “pressure ulcer rate” is routinely reported in the quality reports of all German hospitals. 

Overall, we consider process-based quality indicators as measures that evaluate the dimension of quality with the strongest effects on a patients’ outcome. However, constant modernization of the indicators is the “price” that has to be paid for this [6], [10]. 

The German quality indicators in intensive care medicine have to be seen in the context with other quality-improvement strategies. A strength of the German quality indicators is easy implementation at a given intensive care unit. The process approach does not need any profound change in the local structure, apart perhaps from the willingness of the local stakeholders to change everyday practice. The main intention of the quality indicators remains the adaption of the most relevant therapy processes according to current medical knowledge, so that evidence-based “good practice” is brought to the patient [1]. 

## Application of the quality indicators in intensive care medicine

Evidence based intensive care medicine should be based on consented guidelines and recommendations which are developed according to the most recent literature available. In essence the implementation of the guidelines is the primary goal. To enable this implementation process has to be in the focus in the future. Innovative methods of implementation are needed like “blended learning” concepts, use of multiplier seminars [11] or web-based platforms. Evaluation of guideline implementation requires evaluation of quality indicators. Even if the QI are increasingly used, there are still deficits in widespread application. The reasons for this can be manifold and are presented in Table 1 (Tab. 1) [12]. 

Ideally quality indicators are regularly measured and evaluated for internal quality management. This can’t be achieved everywhere due to a variety of reasons. For example, patient data management systems (PDMS) are not widely used in German intensive care units, which could help technically to collect data for quality measurements. Existing systems do not regularly offer such functions although this would be desired by a majority of its users [13]. Manual computation is more time consuming and calculation may be complex but not an insurmountable obstacle because of the relatively simple calculation rules of the indicators. When beginning data collection for the indicators, a time-limited sample could be useful, particularly since the peer review process is also a method using point prevalence. Retrospective recording of data is also possible. A stepwise approach with an increasing number of indicator use or alternating use of the indicators can be employed. Figure 1 (Fig. 1) shows a process description which would eventually lead to continuous quality improvement in the sense of a PDCA cycle. The effort necessary is not too large compared to the possible positive effects. Moreover, to start the process of recording quality indicators it is recommended to carry out a peer review in intensive care medicine according to the recommendations of the DIVI. 

## The future of DIVI quality indicators

The DIVI quality indicators have grown in importance in recent years. Their value is not limited to the medical processes described. Moreover, the parts of the QI that can be used as structural indicators are also used apart from peer reviews and internal quality management measures. They are used by health insurers for economic considerations (i.e. payment reduction). This has to be seen critical since the indicators have not been developed for such a purpose. Therefore there is a need for a more rigorous formal development process in the future for the indicators [14], [15]. Also the amount of time spent for their development is considerable and it seems appropriate to use external expertise as well. This could be provided by the Working Group of the German Medical Societies (AWMF). This fact is also taken into account by the DIVI. There will be an own sub-section for the quality indicator development located in the section “Quality and Economy”. The peer review process will continue to be the central element of quality assurance in intensive care medicine for the DIVI and the quality indicators developed for it remain one of the main tasks of the DIVI. 

## The third edition of the intensive medical quality indicators for Germany

As in the publication of the first two versions of the QI in intensive care medicine, this third version also includes comments and explanations for each new or revised quality indicator. The list of quality indicators is given in tabular form in the appendix (Attachment 1). Further suggestions that have not been taken into the main quality indicator set for various reasons were in part integrated into the peer review questionnaire to be found online: http://www.divi.de/qualitaetssicherung/peer-review/erste-schritte.html. 

### QI I Daily multiprofessional and interdisciplinary clinical visits with documentation of daily goals

The daily definition of goals in the multiprofessional team consisting at least of medical and nursing staff of the intensive care unit was first described in 2003. The definition of daily goals for the patient in the team improves communication among the treatment team, makes the treatment goals more transparent and increases patient safety with a positive effect on the patient outcome [16]. It is important to involve the multiprofessional team, but also to involve all other clinical partners in the treatment process. Therefore, the indicator was extended by the term interdisciplinary. This does not necessarily imply the simultaneity of visitations. However, the interdisciplinary visitation has to take place in the presence of an experienced ICU physician (in the sense of QI X). The medical documentation, whether paper-based or electronic, must take this into account to ensure the transparency of the daily therapy goals. The achieved goals also need to be documented. Suppliers of documentation products should provide practical solutions with this regard. The further dissemination of electronic patient data management systems (PDMS) is desirable in this context [13].

### QI II Management of sedation, analgesia, and delirium

The QI II in its core is unchanged. By adjusting the title, the focus changes towards a targeted therapy. Numerous scientific publications show an unchanged importance of the subject. In addition to adequate diagnosis and therapy of delirium, patients should practically not be sedated if ever possible and if necessary as short as possible. The recently published S3-guideline is the scientific basis of the indicator [17]. In the indicator a formula for the single topics delirium, analgesia, and sedation is introduced. It is important to record the recommended scales at least every 8 hours. The indicator also demands the presence of a written standard as well as measures of process implementation (i.e. performance indicators). Optionally, the evaluation of an outcome indicator is recommended.

### QI III Patient-adapted ventilation

The indicator has been changed slightly. The evidence for lung-protective ventilation is still high and the implementation in clinical practice is not sufficient. Since there is a tendency to use pressure-controlled ventilation modes in combination with spontaneous breathing, determination of ventilator setting is not valuable in this context. A standard is required for a single ICU which is based on the most recent guidelines by the medical societies [18]. The table with recommended PEEP values in relation to FiO2 was simplified accordingly. In cases of very severe pulmonary failure, cooperation with a specialized center (i.e. for extracorporal lung assist) is recommended [19].

### QI IV Early weaning from invasive ventilation 

This QI has been modified, but the focus remains on termination of mechanical ventilation as quickly as possible. The measures for the prevention of ventilator-associated pneumonia (VAP) previously included in this indicator are now integrated into QI V (“infection prevention”). Overall this indicator also targets VAP prevention using the positive effect on VAP incidence due to a shorter ventilation time. Weaning per se is a very complex process and is closely linked to the sedation concept of an intensive care unit (QI II) and a mobilization concept to avoid neuro-muscular weakness [20]. The latter is the focus of the new QI IX that targets the prevention of atrophy of respiratory muscles as the main pathophysiological factor for weaning failure. The success of this process is linked to well-coordinated standards. A weaning standard is mandatory for structural quality [21].

### QI V Monitoring of infection prevention measures

This indicator is new and is connected to the new QI VI for “infection management measures”. The increasing incidence of multiresistant bacteria and high mortality in the presence of nosocomial infections is the reason for the new indicator [22], [23]. The previous indicator IX (“hand disinfectant consumption”) is now integrated in this indicator. 

Avoiding nosocomial infections is one of the most effective measures to reduce morbidity and mortality in intensive care units [24]. The indicator includes structure, process, and outcome measures. Outcome quality is measured in the formula. Other quality dimensions include structural specifications like appropriate procedural standards for infection prevention [25], [26]. Adequate hand hygiene is the fundamental process of infection prevention. Other infection prevention strategies included in the previous indicator for VAP prevention are still included in the context of bundle use. However, the value of single measures is still under debate. To exemplify this a recent Cochrane review from 2016 considers that oral hygiene (including use of chlorhexidine) reduces the incidence of VAP, but does not influence lethality, length of stay or ventilator days [27]. Effects on lethality were seen for selective digestive decontamination (SDD) [28], [29] and selective oral decontamination (SOD) [30]. To monitor infection prevention hand disinfectant consumption is measured and one of two potential marker infections (VAP or central line-associated bloodstream infections (CLABSI)). The evaluation of both marker infections is useful and recommended.

### QI VI Infection management measures (replaced: Therapeutic hypothermia after cardiac arrest) 

The previous indicator “Therapeutic hypothermia after cardiac arrest” has been replaced. Current guidelines strongly recommend temperature management after resuscitation [31]. In the peer reviews the prevalence is low and therefore does not justify an own indicator. The authors consider it useful to evaluate implementation of temperature management following resuscitation in the peer review questionnaire (see above). 

The new indicator “infection management measures” takes into account the fact that guideline-based therapy of bacterial infections remains a problem. The peer reviews show deficits in correct indication and choice of antiinfective substances as well as correct documentation. Therefore, the indicator focuses on two essential aspects of anti-infective treatment: 1. Timely microbiological testing and 2. Appropriate therapy (indication and adequate) based on recent guideines. 

Clinical signs, which have been redefined in 2016, are the main focus for the early diagnosis of septic shock [32]. Monitoring of clinical signs defined in these publications (level of conciousness, respiratory rate and systolic blood pressure) and the SOFA (sequential organ failure assessment) score are recommended. The second important point is the use of appropriate microbiological tests. This is represented in the indicator formula counting blood cultures per 1,000 patient days [33]. The therapy process which is evaluated e.g. in a peer review focuses on transparent documentation, especially indication and duration of anti-infective therapy. There should be a strong focus on implementation of the determinants of structure and process quality. This includes SOPs [34], timely therapy, multiprofessional visits (microbiologists, clinical pharmacists, infectiologists, etc.), transparent documentation, therapeutic drug monitoring [35], and antibiotic stewardship (ABS) [36]. 

### QI VII Early enteral nutrition

This indicator was slightly changed. In recent years, numerous publications on the topic of nutrition in ICU patients have been published. In the most recent guideline of the American Society (ASPEN) clear recommendations on enteral nutrition are available [37]. The current guidelines of the European Society (ESPEN), which are roughly 10 years old, particularly favor an earlier start of nutrition therapy [38], [39], especially with regard to the start of parenteral nutrition to achieve adequate caloric intake. The goal of the indicator takes this guideline and the more recent literature into account and recommends a minimum caloric intake after 48 hours. Overall, early enteral nutrition remains the main goal [40]. In addition to offer nutrition in a “natural” way, the adequate nutrient composition and energy supply for the patient must be considered. It is strongly recommended to define patient-specific nutritional targets and to establish a nutrition standard for an ICU [41]. 

### QI VIII Documentation of structured patient and family communications 

This indicator has minor changes. The focus has been shifted towards the determination of a patient’s advance directives [42]. The indicator additionally recommends measures for the wellbeing of relatives and staff. The evaluation of peer reviews show documentations of family discussions are not sufficient. The documentation often does not reveal what subjects were discussed and what consensus was reached with regard to therapeutic goals that are in the patient’s best interest. The demand for adequate documentation templates remains. Intensive care diaries and the evaluation of family surveys are potentially important complementary measures. The DIVI has published recommendations on adequate therapy in end-of-life situations [43], [44]. 

### QI IX Early mobilization (replaces “Hand disinfectant consumption”)

The indicator “Early mobilization” was added to the quality indicators. As mentioned above the QI IX (“Hand disinfectant consumption”) became part of the new QI V (“infection prevention”). 

With an increasing numbers of long-term ICU patients suffering chronic critical illness, any measure is useful to avoid long-term dependence on respiratory support (see also QI II and IV). The positive effect of early mobilization on ICU patients has been demonstrated in various publications [45], [46], [47], [48], [49]. The indicator emphasizes on the early start of mobilization measures, which must be defined in local standards [50]. The safety of mobilization measures is sometimes doubted. National and European recommendations regarding safety can be used to establish local standards [51]. In addition, avoiding immobilization is also important. If immobilization is indicated it should be ordered explicitly. 

### QI X Direction of the intensive care unit 

This indicator has been slightly changed. The evidence on the content of this indicator is still clear. The 24-hour presence of an experienced and qualified team of nurses and physicians is necessary to ensure adequate care for ICU patients. The term presence still allows the ICU physician to be part of emergency teams (NET, Resuscitation Service) working shortly outside the ICU. This does not include other clinical obligations outside the ICU or the hospital. During the core working time, i.e. [52], [53] during the time when important therapeutic decisions are made (in the interdisciplinary context) the availability of all "decision-makers" is necessary, especially the presence of a specialist intensivist (= experienced and qualified intensive care physician). This experienced and qualified intensivist may not have relevant clinical obligations except responsibility for the ICU. In order to fulfill the indicator, the structural requirements of the DIVI for an ICU have to be followed [54]. This includes adequate staffing, which is ensured by close contact between lead physicians, nursing managers, and hospital management. Staffing is adapted to structural demands that may be different in single institutions. They may depend on external services provided like dialysis service, patient transport or maintenance of equipment and pharmacy service.

## Notes

### Competing interests

The authors declare that they have no competing interests regarding the content of this manuscript.

## Supplementary Material

Quality Indicators Intensive Care (3rd Edition 2017)

## Figures and Tables

**Table 1:**
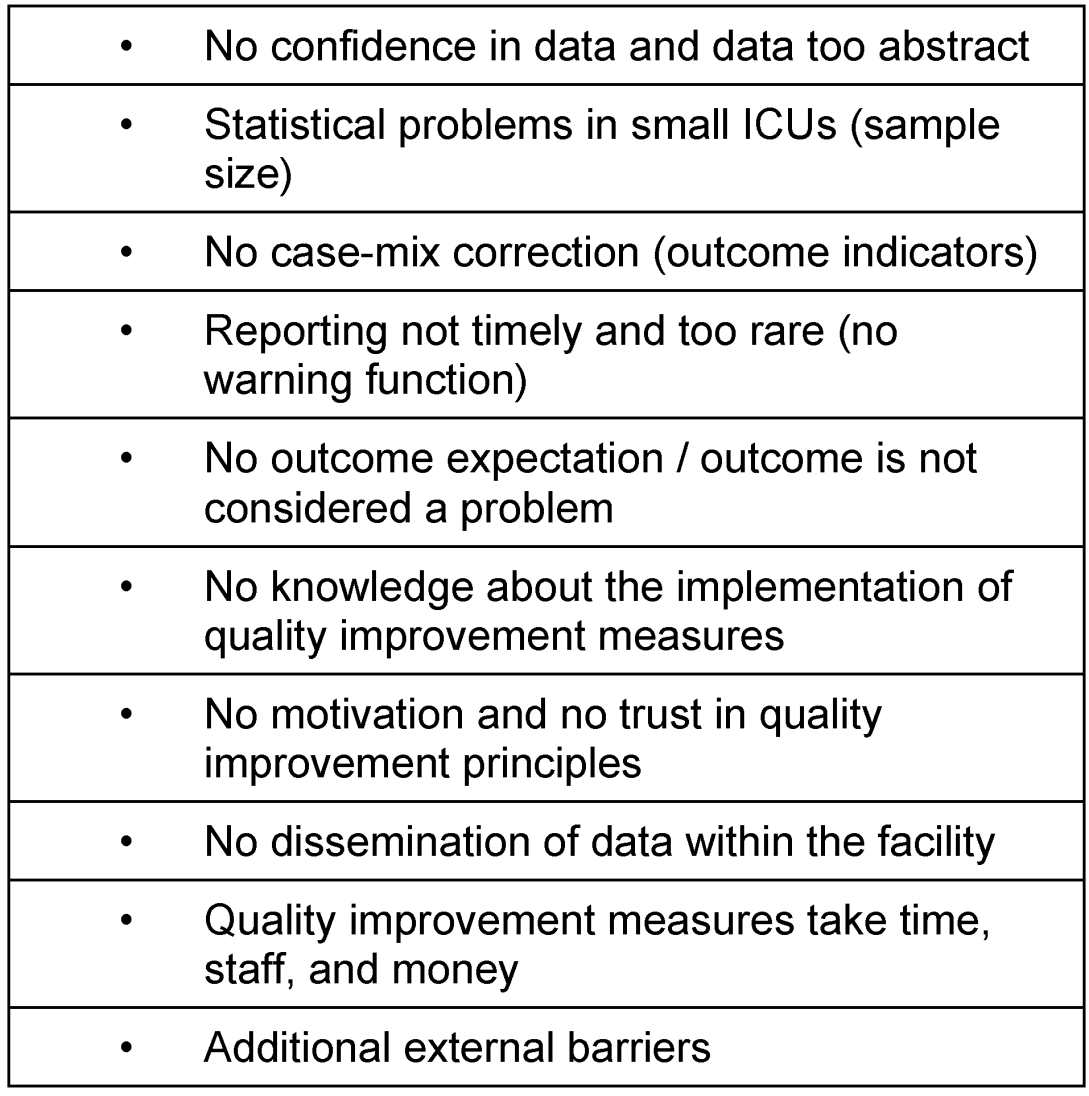
Obstructing factors for QI implementation (according to de Vos et al.) [12]
