# Supplementary material for: Quality indicators in intensive care medicine for Germany – third edition 2017
Source: Ger Med Sci. 2017 Aug 1;15:Doc10. doi: 10.3205/000251 (PMC5541336; doi:10.3205/000251)
Supplement: Quality Indicators Intensive Care (3rd Edition 2017) [file GMS-15-10-s-001.pdf]

Attachment 1

Quality Indicators Intensive Care (3rd Edition 2017)

Number	**Quality Indicators I–X**
**I**	**Daily multiprofessional and interdisciplinary clinical visits with documentation of daily goals**
**II**	**Management of sedation, analgesia and delirium**
**III**	**Patient-adapted ventilation**
**IV**	**Early weaning from invasive ventilation**
**V**	**Monitoring of infection prevention measures**
**VI**	**Measures for infection management**
**VII**	**Early enteral nutrition**
**VIII**	**Documentation of structured patient and family communication**
**IX**	**Early mobilization**
**X**	**Direction of the intensive care unit**

Quality Indicators Intensive Care (2nd Edition 2013)

Number	**Quality Indicators I–X**
**I**	**Daily multiprofessional ward rounds with the documentation of daily therapy goals**
**II**	**Monitoring sedation, analgesia, delirium**
**III**	**Lung protective ventilation**
**IV**	**Weaning and other measures to prevent ventilator associated pneumonias**
**V**	**Early and adequate initiation of antibiotic therapy**
**VI**	**Therapeutic hypothermia after cardiac arrest (CA)**
**VII**	**Early enteral nutrition**
**VIII**	**Documentation of structured relative- / next-of-kin communication**
**IX**	**Hand disinfectant consumption (BQS Indicator 2010)**
**X**	**Direction of the ICU by a specialist dedicated intensivist with no other clinical duties in a department. Presence of a specialist ICU-physician during daytime and presence of experienced intensive care physicians and nurses over the course of 24 hours a day.**

Main indicator I

Name of the indicator	**Daily multiprofessional and interdisciplinary clinical visits with documentation of daily goals**
Dimension	Risk and effectiveness
Justification	The daily multiprofessional and interdisciplinary ward round improves the communication of the professions involved in the treatment on an intensive care unit, in particular the medical and nursing team members. Of particular importance is the written documentation of the daily goals for each patient in the intensive care unit. The determination of daily (short-term) and long-term goals helps to improve treatment quality and to avoid complications. It leads to more effective implementation of planned measures.
Formula	$\frac{Documented\ daily\ rounds\ with\ definition\ of\ therapy\ goals}{Treatment\ days} \times 100$
Population	All patients in the ICU
Explanation of terminology	**Ward round:** Interprofessional and - depending on the treatment spectrum of the intensive care unit - also interdisciplinary case discussion with at least one decision maker (chief physician, head of the intensive care unit) present. The ward rounds should enable all participating professions to provide and receive information regarding the patient's clinical picture. Interruptions of rounds and thus interruptions of this flow of information should be minimized as far as possible.<br>**Daily goals:** The daily goals should be defined during the round, taking into account all professions and disciplines involved.<br>The following points may be focused on when defining daily goals:<br>• Coordinating communication (consultations / relatives / others persons taking part in treatment)<br>• Therapeutic goals/change in therapeutic goals<br>• Targets for analgesia, sedation and delir management<br>• Ventilation/weaning/respiratory therapy<br>• Circulation/fluid homeostasis<br>• Nutrition<br>• Infection management<br>• need for catheters and other invasive procedures<br>• Definition of prevention measures (anticoagulation / decubitus / gastric protection / mobilization / special physiotherapy measures)<br>• Planned measures (diagnostic / therapeutic)<br>• Agreement over medication<br><br>**Documentation:** The more professions or disciplines are involved in the treatment of a patient, the more difficult it is to unite participants synchronously to a visit. Therefore, written specifications are of utmost importance in order to guarantee the flow of information. Written documentation to show what has been defined by whose participation helps those who have primarily not attended the round, to comprehend what was considered important. Changes in therapeutic goals can easier be followed.<br>The communication-enhancing effect of a multiprofessional and inter-disciplinary round can be supported by special document templates that can be inserted into the daily documentation sheets. Daily goal

	checklists support the implementation of daily goals as has been shown in the literature. However, checklists alone do not improve patient safety. Improving communication and dissemination of information in intensive care units is a complex challenge for all involved in the ICU. Awareness for this problem should be cultivated.
Type	Structure / Process
Source of data	Clinical records / PDMS
Standard	1. Structure: Standard yes / no; Yes = 100%<br>2. Process: Implementation yes / no; Yes >70%
Literature	[16, 55-60]

Main Indicator II

Name of the indicator	**Management of sedation, analgesia and delirium**
Dimension	Risk and effectiveness
Justification	Inadequate sedation (over or undertherapy), inadequate analgesia and untreated delirium cause prolonged mechanical ventilation, prolonged intensive care stay, prolonged hospital stay and increase morbidity, mortality and resource utilization. The use of validated sedation, analgesia and delirium scores is recommended in clinical guidelines.<br>The indicator is divided into the management of sedation, analgesia and delirium, respectively:<br>Evaluation of structure: Are there SOPs that cover all three subjects (sedation, analgesia and delirium)?<br>and evaluation of the process focusing on the question how often are individual scores collected.<br>Optional measurement of outcome quality (an analysis is recommended at least once per year. institutions using a PDMS may use shorter intervals.)<br>A) sedation (periods without sedation, times in the target range +/- 1)<br>B) Analgesia (percentage of pain score in target range)<br>C) Delir (only prevalence assessment, was a therapy initiated, if yes, what therapy?)
Formula	Generally applicable for sedation, pain and delirium. All dimensions are measured.<br>$\frac{\text{Number of all performed measurements [SCORE])}}{\text{Total number of measurements} *} \times 100$<br>* Total number of predefined measurements = (treatment days-1) x3
Population	All ICU patients throughout the treatment period

<table>
<tr><td>Explanation of terminology</td><td>
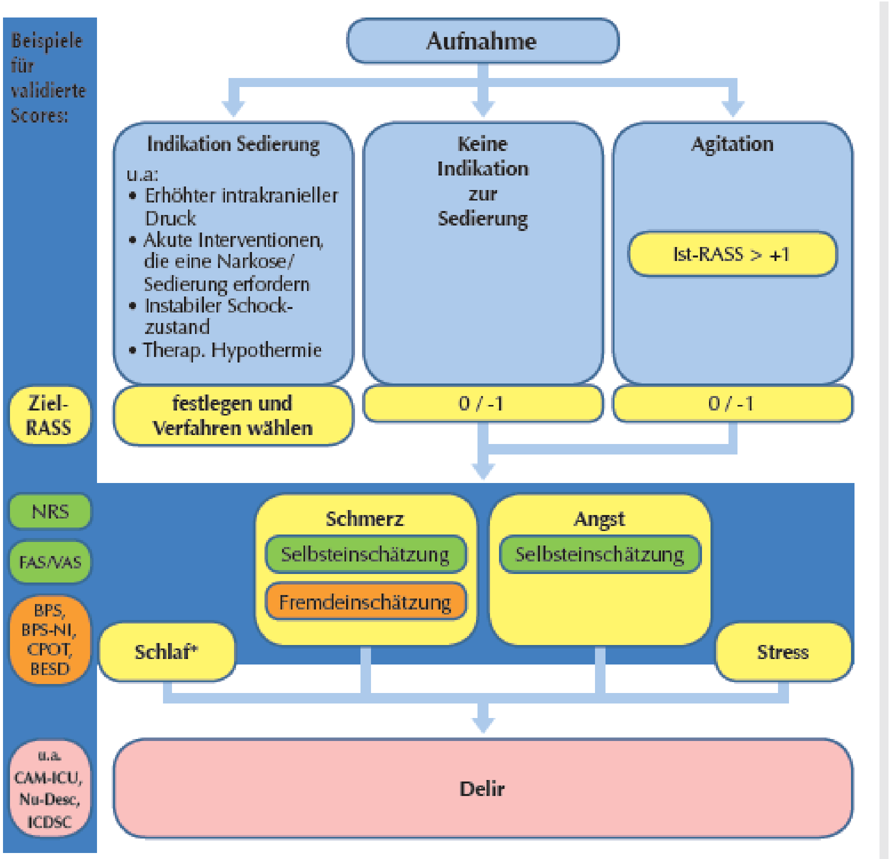

Implementierungshilfe für das DAS-Management mit Beispielen für validierte Messskalen: **RASS:** Richmond Agitation-Sedation Scale, **NRS:** Numerische Rating-Skala, **VAS:** Visuelle Analog-skala, **BPS:** Behavioral Pain Scale, **BESD:** Beurteilung des Schmerzes bei Demenz, **FAS:** Faces Anxiety Scale, **CAM-ICU:** Confusion Assessment Method for the Intensive Care Unit, **ICDSC:** Intensive Care Delirium Screening Checklist, **Nu-Desc:** Nursing Delirium Screening Scale; *Polysomnographie: validiert, aber zu aufwändig, Schlaf-Überwachungsapps verfügbar, im ICU-Setting nicht validiert.

(Algorithm from Wolf A., et al. Anästh Intensivmed 2016;57:41-44)

Monitoring: assessment of the sedation and analgesia level
as well as the
presence of delirium based on validated scales every 8 hours or when the clinical situation changed.
</td></tr>
<tr><td>Type</td><td>Structure / Process</td></tr>
<tr><td>Source of data</td><td>1. Structure: Query
2. Process: patient records; PDMS
3. Outcome: patient records; PDMS</td></tr>
<tr><td>Standard</td><td>1. Structure (SOPs: sedation / analgesia / delirium) Default yes / no (yes = 100%)
2. Process scoring (sedation / analgesia / delirium): Frequency of scoring ≥70%
3. Result (optional): Target / Actual Comparison (Sedation / Analgesia / Delirium) (No specifications)
Recommended scales [SCORE]:
RASS: Richmond Agitation and Sedation Scale
NRS: Numeric Rating Scale and BPS: Behavioral Pain Scale
CAM-ICU: Confusion Assessment Method - Intensive Care Unit
ICDSC: Intensive Care Delirium Screening Checklist
or other validated delirium scores</td></tr>
<tr><td>Literature</td><td>[17, 61-64]</td></tr>
</table>

Main Indicator III

<table>
<tr><td>Name of the indicator</td><td>Patient-adapted ventilation</td></tr>
<tr><td>Dimension</td><td>Risk and effectiveness</td></tr>
<tr><td>Justification</td><td>Patient-adapted ventilation strategies were able to reduce ventilator-associated injury and improve outcome of mechanically ventilated patients. A standardized concept for ventilation therapy is useful and should be maintained. In severe pulmonary failure and the failure of maximum conservative treatment measures (prone position, muscle relaxation, lung recruitment maneuvers), it may be necessary to contact a specialized treatment center to establish an extracorporeal lung assist.</td></tr>
<tr><td>Formula</td><td>$\frac{Days\ on\ standardized\ mechanical\ ventilation\ according\ to\ QI\ protocol}{Total\ mechanical\ ventilation\ days} \times 100$</td></tr>
<tr><td>Population</td><td>All mechanically ventilated patients
All days of mechanical ventilation over total treatment period</td></tr>
<tr><td>Explanation of terminology</td><td>Evident ventilation goals are ventilation with low tidal volumes and low peak pressure:
1. With controlled ventilation: 6 ml / kg predicted body ideal weight (this is not applicable for assisted ventilation modes)
2. Recommended PEEP setting in relation to FiO2
<table>
<tr><td>FiO2</td><td>Up to 0.4</td><td>0.4-0.5</td><td>0.5-0.6</td><td>0.6-0.7</td><td>0.7-0.8</td><td>0.8-0.9</td><td>1.0</td></tr>
<tr><td>PEEP</td><td>5</td><td>8</td><td>10</td><td>10-14</td><td>14-16</td><td>16-18</td><td>18-24</td></tr>
</table>
The values given in the table have to be regarded as starting values or reference values and should be adapted as required. The influence of the PEEP setting on the following parameters has to be considered: oxygenation, driving pressure, transpulmonary pressure, hemodynamics (specific function of the right ventricle.)
3. Plateau pressure <30 cm H2O</td></tr>
<tr><td>Type</td><td>Structure / Process</td></tr>
<tr><td>Source of data</td><td>1. Structure: Standard yes/no; Tested yes/no
2. Step: Peer Review, Clinical records/PDMS</td></tr>
<tr><td>Standard</td><td>1. Structure Yes 100% (SOP – ventilation standard)
2. Process: ≥70% patient-adapted ventilation</td></tr>
<tr><td>Literature</td><td>[18, 19, 65-79]</td></tr>
</table>

Attachment to: Kumpf O, Braun JP, Brinkmann A, Bause H, Bellgardt M, Bloos F, Dubb R, Greim C, Kaltwasser A, Marx G, Riessen R, Spies C, Weimann J, Wöbker G, Muhl E, Waydhas C. Quality indicators in intensive care medicine for Germany – third edition 2017. GMS Ger Med Sci. 2017;15:Doc10. DOI: 10.3205/000251

Main Indicator IV

Name of the indicator	**Early weaning from invasive ventilation**
Dimension	Risk and effectiveness
Justification	Invasive ventilation is associated with the risk of ventilator-associated pneumonia (VAP) and other possible complications. Therapeutic goal is therefore to start efforts to discontinue mechanical ventilation as soon as possible (so called "weaning") if disease severity allows this. Depending on the type and severity of the disease, it should also be examined whether invasive ventilation can be completely avoided by the appropriate application of non-invasive ventilation (NIV) or by the application of oxygen via high-flow nasal canula (HFNC) or if reintubation after primary successful extubation can be prevented.
Formula	$\frac{Number\ of\ invasively\ ventilated\ patients\ with\ documentation\ of\ weaning\ capability\ or\ weaning\ trial}{Total\ \text{time of all mechanical ventilation days}} \times 100$
Population	All patients with mechanical ventilation<br>All days of mechanical ventilation over total treatment period
Explanation of terminology	Weaning protocol / concept in combination with sedation targets:<br>For every ventilated patient, the potential for weaning should be evaluated daily or a weaning trial should be carried out. The use of standardized weaning protocols can improve the results. There is also a close connection with the QI II, which specifies the targets of sedation and the documentation of collected score values.
Type	Structure / Process
Source of data	Structure: Peer Review<br>Existence of a weaning concept<br>Process: patient records, PDMS, peer review<br>Ward round: Indication for invasive ventilation present yes / no,<br>Daily documentation of ventilation targets / weaning:<br>Process: Indication for a NIV or HFNC<br>Yes / no (patient record, PDMS, peer review),
Standard	Structure Yes / No (Yes = 100%)<br>Process: >70% number of positive responses, Missing Values <20%
Literature	[20, 21, 80-86]

Main Indicator V

Name of the indicator	**Monitoring the measures for the prevention of infection**
Dimension	Risk and effectiveness
Justification	Patients in the intensive care unit have a high risk of hospital acquired infections. Intensive care units therefore belong to the high-risk area. This is becoming more and more important with the increasing occurrence of multiresistant pathogens (MRE). Within the framework of the Infection Protection Act (Infektionsschutzgesetz), medical institutions have a high degree of responsibility in the prevention of those infections.<br>1**. Structure quality:**<br>To implement an effective infection prevention, established hygiene rules must be followed. These rules cover many areas of medical activities in the intensive care unit (for example: hand disinfection, dealing with patients carrying multiresistant pathogens, VAP prophylaxis, hygienic measures for invasive procedures). The necessary measures should be documented as standard operating procedures (SOPs) for infection prevention.<br>2**. Process quality:**<br>Adequate hand hygiene is a fundamental component of the prevention of nosocomial infections. Therefore the German campaign "Action Clean Hands" was launched on the basis of the WHO campaign "Clean Care is Safer Care" to improve the compliance to hand disinfection rules. This compliance can be monitored indirectly by measuring hand disinfectant consumption.<br>3**. Outcome quality**<br>Ventilator-associated pneumonias (VAP) and Central Line-Associated Bloodstream Infections (CLABSI) are typical infectious complications in the intensive care unit, for which prevention recommendations exist. Monitoring the frequency of at least one of these infections (Surveillance) offers the possibility to identify problems in hygiene management and to evaluate the success of quality-improving measures.
Formula	The quality indicator infection prevention monitors outcome quality as a measure for implementation of infection prevention.<br>Type and number of nosocomial infections<br>(Selection of a marker infection: VAP and/or CLABSI)<br>The incidence of nosocomial infections should be stated normalized for 1000 application days:<br>• VAP frequency per 1000 invasive ventilation days<br>• CLABSI frequency per 1000 central venous line day<br>• Hand disinfectant consumption
Population	All patients in the intensive care unit<br>Staff of intensive care unit
Explanation of terminology	**Possible measures for VAP prevention:**<br>For VAP-prevention, various measures are known from the literature, which either as a bundle of measures (VAP Bundle) or as a single measure contribute to the reduction of VAP incidence. The composition of a VAP bundles differ in the literature so that, given the positive outcome-relevant effect of the bundle, there can be no clear assignment

	to only one of the mentioned measures. VAP bundles as such are, however, well-suited to reduce the incidence of VAP. It is recommended to have at least three measures of a VAP bundle present in the SOPs of the intensive care unit, e.g.: Oral Care<br>Avoidance of pulmonary aspiration e.g. by cuff pressure measurements, subglottic secretion suction or oral antiseptic solutions. There are conflicting data on safety regarding the use of oral chlorhexidine. The use of antibiotics for selective oral decontamination (SOD) and selective digestive decontamination (SDD) should prompt to evaluate local data on bacterial resistance.<br><br>**Possible measures for CLABSI prophylaxis**<br>It is recommended to define a standard procedure for the insertion and maintenance of intravascular catheters and to train its use. Measures for the insertion of the catheters should include: hand disinfection before puncture, recommendation on the choice of skin disinfection (eg chlorhexidine-containing solutions), maximum sterile barrier precaution (sterile gloves, sterile gown, mask, sufficiently large sterile coverage), recommendations regarding puncture techniques (Avoidance of the femoral vein as a puncture site, use of sonography). Measures for catheter care should include recommendations on disinfection when using the catheter, indications for use (avoiding unnecessary manipulation), quick removal of the catheter and care of the insertion site.<br><br>**The 5 Indications of hand disinfection:**<br>1. BEFORE patient contact<br>2. BEFORE an aseptic activity<br>3. AFTER contact with potentially infectious materials<br>4. AFTER patient contact<br>5. AFTER contact with the immediate patient environment
Type	Outcome
Source of data	Patient files or KISS data<br>Management data (Disinfectant consumption)
Standard	Outcome:<br>• Declining rate(s) of nosocomial infections over time with the selected marker infection(s)<br>• Hand disinfectant consumption >80 - 100 liters / 1000 patient days
Literatur	[22-30, 87-106]

Main Indicator VI

Name of the indicator	**Measures for infection management**
Dimension	Risk and effectiveness
Justification	Early, adequate and effective infection diagnosis and anti-infective therapy as well as the effective prevention of bacterial resistance are of paramount importance in the management of infections in the intensive care unit. The following principles should be followed:<br>1. Early and adequate, calculated antibiotic therapy in patients with severe infections and organ failure (sepsis and septic shock). In patients with low disease severity differentiated diagnostic measures and targeted therapy.<br>2. Adequate microbiological testing before the start of antibiotic therapy<br>3. Measures to avoid unnecessary anti-infective treatment<br><br>In addition to source control, qualitatively and quantitatively adequate microbiological testing and adequate anti-infective therapy is crucial for the survival of critically ill patients with severe infections.<br><br>Integrals and indispensable determinants in the sense of process quality are:<br>• Adherence to guidelines<br>• Early and adequate microbiological diagnostics before beginning therapy (SOP)<br>• Timely (<1 h) and adequate anti-infective therapy (SOP)<br>• Multiprofessional visitations (intensive care nurses and physicians, infectious disease specialist (if available), hygiene specialist, microbiologist, clinical pharmacist)<br>• Transparent documentation of indication and duration of anti-infective therapy<br>• Use of therapeutic drug monitoring (TDM, especially for aminoglycosides and glycopeptides)<br>• Use of Antibiotic Stewardship (ABS)
Formula	$\frac{Number\ of\ blood\ cultures}{1000\ patient\ days}$<br><br>$\frac{Number\ of\ adequate\ antiinfective\ treatment}{Overall\ number\ of\ patients\ with\ antiinfective\ therapy} \times 100$<br><br>A. Indication<br>B. Documentation<br>C. Start <1 hour after diagnosis
Population	All patients with DRG code.
Explanation of terminology	The recently formulated sepsis definition from 2016 focuses on clinical aspects. In addition, the SOFA score is thought to be important in the detection of sepsis. It is recommended to record the components of the SOFA score daily in all patients with relevant sepsis risk. For current definition of sepsis and septic shock, please refer to The Third International Consensus Definitions for Sepsis and Septic Shock (Sepsis-3), JAMA, 2016, (JAMA. 2016;315(8):801-810. doi:10.1001/jama.2016.0287

	http://jama.jamanetwork.com/article.aspx?articleid=2492881)
Type	Process and outcome
Source of data	1. Hospital information system, PDMS<br>2. Intensive KISS (NRZ)<br>3. Routine DRG data<br>4. Modular Certificate of Intensive Care Units (DGAI)<br>5. Peer Review in Intensive Care
Standard	1. Number of blood cultures ≥100/1000 patient days<br>2. Number of adequate antibiotic therapy >80%
Literatur	[32-36, 107-124]

Main Indicator VII

Name of the indicator	**Early Enteral Nutrition**
Dimension	Risk and effectiveness
Justification	The early start of enteral nutrition (EN) within the first 48 hours is associated with a reduction of infectious complications and a lower mortality rate of intensive care patients. There is sufficient consensus in the ASPEN recommendation on the appropriate calorie quantity. The definition of a calorie goal is strongly recommended. Parenteral nutrition as a supplement can be useful to achieve the desired calorie goal.
Formula	$$\frac{Number\ of\ adequatelly *\ enterally\ fed\ patients}{\text{number of patients that can be fed enterally}} \times 100$$ (* At least 50% of the daily recommended calorie intake)
Population	All patients in the intensive care unit
Explanation of terminology	Indication for EN: All patients without contraindication for enteral nutrition, who do not tolerate a complete oral diet. The calorie goal is based on age, body weight and nutritional status of the patient. The current practice guidelines show no consensus on the appropriate amount of energy . At least 50% of the daily nutrition requirement should be reached within 48 hours. Nutritional therapy should be done according to a standard. An early enteral diet avoids a calorie deficit in the patient, which has a negative effect causing increased infection rates and an extended length of stay. It is recommended to use nutritional protocols to establish early enteral feeding. Supplementary parenteral nutrition can close the gap between the patient's calorie requirements and the enterally supplied energy. The enteral and metabolic tolerance of the patient should be taken into account. The European guidelines recommend an additional parenteral diet if the calorie target cannot be achieved by enteral nutrition after 3 days. They define about 25 kcal / kg / d as the calorie target. A strict adjustment of the blood sugar can no longer be generally recommended. New investigations favor an upper limit of blood glucose levels of 10mmol / l or 180 mg / dl.
Type	Structure: Yes / no (SOP available)<br>Process: Number of appropriately fed patients<br>Process: Start within the first 48 hours (implementation rate)
Source of data	Query<br>Process: Patient / PDMS, Peer Review<br>Process: Patient / PDMS, Peer Review
Standard	Structure: 100%<br>Process: ≥70%<br>Process: ≥70%
Literature	[37-41, 125, 126]

Main Indicator VIII

Name of the indicator	**Documentation of a structured patient and family communication**
Dimension	Risk and effectiveness
Justification	The determination of the patient's preferences is of utmost importance in the planning and implementation of intensive care treatment. Only the interplay of correct medical indications and the determined patient's preferences leads to appropriate therapy decisions and avoids conflicts with patients and family members. The communication of therapeutic goals between intensive care personnel and patients and relatives helps building trust but also reduces grief and grief-induced morbidity (depression, PTSD) in patients and their families. It also serves to prevent staff morbidity.<br>In order to make communication results sustainable, their documentation is mandatory. For the coping with the critical illness of a family member the use of a patient diary can be helpful. Quastionnaires for patient and / or family satisfaction help adjust these communication processes if necessary.
Formula	$\frac{\text{Appropriately documented communications}}{\text{Documented communications}} \times 100$
Population	All patients in an intensive care unit after a critical event
Explanation of terminology	Documentation of family discussions of patients after a critical event in an intensive care unit. A critical event is an emergency or the unplanned admission or a sudden change in the patients condition.<br>Within 48 hours after the event and furthermore at least once a week, a communication should be documented whose content meets the following requirements:<br>1. Explanation of the patient's current status<br>2. Current treatment plan<br>3. Determination of the patient's wishes either through the patient himself or through his relatives. Determination of the perspective of the family, provided the patient can not speak freely for himself.<br>4. To what extent can patient preferences and therapeutic goals be brought into concordance?<br>5. Indication of short-term, medium-term targets / prognosis determined by the treatment team<br>6. Conclusion / Definitions / Consequences<br><br>A detailed presentation of aspects of these discussions is given in the position papers of the DIVI from 2006 and 2012. Each discussion should be documented with the names of the participants (including representatives of the interprofessional treatment team) and date.
Type	Structure and process
Source of data	Structure: Query<br>Process: Patient file / PDMS, Peer Review<br>Process: Patient file / PDMS, Peer Review
Standard	Structure: 100%<br>Process: ≥70%<br>Process: ≥70%

	Structure: documentation template available Yes = 100%<br>Process: First conversation within 48 hours after admission and least one conversation per week thereafter: Yes >70%<br>Process: Correct formal and content documentation: Yes >70%
Literature	[42-44, 127-141]

Main Indicator IX

Name of the indicator	**Early Mobilization**
Dimension	Risk and effectiveness
Justification	Early mobilization describes measures for the prophylaxis or therapy of long-term negative consequences of immobilization on pulmonary, muscular and cognitive functions.<br>The quality indicator includes:<br>1. Presence of standard operating procedures<br>2. Implementation of these standards<br><br>Aims of Early-mobilization, adjuvant to intensive care treatment, are an improved pulmonary function of the patient by e.g. improved clearance of secretion, and the maintenance and improvement of muscular function. Likewise, the cognitive function is positively influenced. The result is a significantly shorter length of stay in the intensive care unit and in the hospital. In the long term, the aim of early mobilization is to achieve greater independence in daily activities of the patient (ADL).<br>Possible forms of positional changes with proven favorable effects are:<br>• Prone position<br>• Side bearing<br>• Sitting position<br>• Half-sitting position<br>• Anti-Trendelenburg position<br><br>Unfavorable changes in position are:<br>• Shallow back<br>• Trendelenburg position<br>Early-mobilization means the mobilization of the patient within the first 72 hours or earlier. These includes passive, assisted-active and active measures. It is recommended to integrate measures for early mobilization into an overall treatment concept and to create a standardized algorithm which is then implemented in a patient-adapted manner. In addition, it is recommended to order medically necessary immobilization explicitly.
Formula	$\frac{\text{Number of patients who have undergone early mobilization}}{\text{number of patients who have met the criteria for early mobilization}} \times 100$<br><br>Total of immobilized patients without explicit medical order = 0
Population	1. All invasively ventilated patients<br>2. All patients in the intensive care unit
Explanation of terminology	Early mobilization is achieved by close cooperation of nursing specialists with physiotherapists. Early-mobilization depends on an adequate staffing. The criteria to begin early mobilization should be documented. In this context, consensus recommendation can be helpful in which the safety of mobilization measures is described depending on the invasive therapeutic methods used.
Type	Structure and process
Source of data	1. Query, peer review<br>2. Patient records, PDMS, care documentation

Standard	Structure: Presence of an algorithm for Early mobilization<br>Standard or SOP/Algorithm available? Yes NoYes = 100%<br>Process: (implementation) Immobilization is ordered explicitely<br>Implementation yes/no<br>Yes >90%<br>Total of immobilized patients without order = 0
Literature	[45-51, 142-147]

Main Indicator X

Name of the indicator	**Direction of the intensive care unit**
Dimension	Appropriateness, Risk and effectiveness
Justification	The management of the intensive care unit by a certified intensivist which has no other clinical obligations, the presence of a certified intensivist during the core working period and presence of intensive care physicians and nurses staff over 24h ensures the quality of the care and reduces mortality and treatment duration of ICU patients. High-quality care of ICU patients requires the presence of experienced staff around the clock. Management level nurses and physicians as well as hospital administration have together with hospital-management to ensure the implementation of the personnel requirements of the DIVI.
Formula	$\frac{\text{Number of days with fulfillment of structural specifications}}{365} \times 100$
Population	All days of the year over the observed period
Explanation of terminology	Personal presence of certified intensivist in the core working time is considered necessary. In the literature, outcome-relevant structural specifications corresponding to the QI X can be found. The intensive care unit has to be staffed with medical and nursing staff who is not assigned other obligation and who is aware of the current problems of the patients.
Type	Structure
Source of data	Staff department and duty roster
Standard	97% of the days
Literature	[52-54, 148-151]
